# Feasibility, Method and Early Outcome of Image-Guided Volumetric Modulated Arc Radiosurgery Followed by Resection for AJCC Stage IIA–IIIB High-Risk Large Intraocular Melanoma

**DOI:** 10.3390/cancers14194729

**Published:** 2022-09-28

**Authors:** Maja Guberina, Ekaterina Sokolenko, Nika Guberina, Sami Dalbah, Christoph Pöttgen, Wolfgang Lübcke, Frank Indenkämpen, Manfred Lachmuth, Dirk Flühs, Ying Chen, Christian Hoffmann, Cornelius Deuschl, Leyla Jabbarli, Miltiadis Fiorentzis, Andreas Foerster, Philipp Rating, Melanie Ebenau, Tobias Grunewald, Nikolaos Bechrakis, Martin Stuschke

**Affiliations:** 1 Department of Radiotherapy, University Hospital Essen, West German Cancer Center, University Duisburg-Essen, Hufeland Str. 55, 45147 Essen, Germany; 2 German Cancer Consortium (DKTK), Partner Site University Hospital Essen, Hufeland Str. 55, 45147 Essen, Germany; 3 Department of Ophthalmology, University Hospital Essen, University of Duisburg-Essen, Hufeland Str. 55, 45147 Essen, Germany; 4 Institute of Diagnostic, Interventional Radiology and Neuroradiology, University Hospital Essen, University Duisburg-Essen, 45147 Essen, Germany

**Keywords:** image-guided, volumetric modulated arc radiotherapy, radiosurgery, endoresection, transcleral resection, large intraocular melanoma, outcome

## Abstract

**Simple Summary:**

The aim of this trial was to define one optimal contemporary treatment procedure for large intraocular melanoma. Radiosurgery is a highly effective treatment in cancer. In this trial, all consecutive patients with large intraocular melanoma treated with multimodality treatment, comprising 4D image-guided volumetric modulated arc radiosurgery procedure followed by resection, were evaluated. In the short-term follow-up there was no clinical toxicity due to external beam radiation therapy, and no local tumor recurrence. In 98% of the cases, the eye bulb could be maintained with partial residual visual acuity in the mean follow-up of 18 months. The outcome estimates one optimal treatment procedure for high-risk, large intraocular melanoma, with excellent results in the first follow-up.

**Abstract:**

The main objective of this prospective observational study was the characterization of the feasibility and early outcome of image-guided (IG) volumetric modulated arc (VMAT) radiosurgery (SRS) followed by resection for patients with large intraocular melanoma. Our study included consecutive patients with unfavorable-risk melanoma, enrolled in an ophthalmic oncology center. IG-VMAT-SRS was applied by high-resolution 4D image guidance and monitoring. Current stereotactic technique parameters were evaluated for comparison. Side effects and eye function, based on a 5-point CTC assessment score, were quantified. In patients with tumors located more than 0.7–1 mm apart from the optic nerve, partial to complete volume-sparing of the optic nerve head could be achieved. In 95.5% of this subgroup, the vitality of the optic nerve and vision could be preserved by the multimodality-treatment approach (mean follow-up: 18 months (7.5–36 months)). The advanced technology of stereotactic radiotherapy demonstrated the achievability of steep dose gradients around the high-dose volume, with 4D-IG-VMAT dose application. These results enforce IG-VMAT-SRS followed by resection as one of the major therapeutic options for patients with large intraocular melanoma. The combination of 4D-IG high-precision SRS and resection provides an effective treatment for large intraocular melanoma, with few side effects, and enables an eye bulb and even vision preserving modus operandi.

## 1. Introduction

The optimal treatment for large intraocular melanoma depends on the parameters of the primary tumor, eye impairment and visual acuity, staging and patient comorbidities. Patients with favorable-risk intraocular melanoma can be adequately treated with brachytherapy alone [1], whereas patients with large, unfavorable-risk intraocular melanoma (clinical AJCC stage IIA–IIIB, cT2a/b–cT4a/b cN0 cM0) require more intensive treatment. Formerly, the primary treatment for large intraocular melanoma was enucleation.

At present, external beam stereotactic photon radiotherapy or proton therapy are among the main treatment options in these cases [2]: long-term follow-up indicates very good local tumor control, in patients with large intraocular melanomas treated with fractionated proton irradiation, of about 92% at 5 years. But visual acuity diminished steadily after treatment. Vision of 20/200 or better was maintained in only 23% of patients after 3 years, and 16% after 5 years [2]. Moreover, in 22–38.5% of patients with large tumors, the eye bulb could not be durably preserved, due to tumor and therapy-related factors. The causes can be manifold. The main leading complications are toxic tumor syndrome (TTS), retinal detachment (RD) or neovascular glaucoma (NVG) [3].

Thus, there have been further studies analyzing combined treatment modalities, including radiotherapy followed by eye-preserving surgery (endoresection or transscleral resection). The main rationale is the use of preoperative radiosurgery in the no-touch technique for tumor devitalization and maximum protection of the main visual axis [4,5]. Consequently, the residual mass can be eliminated with minimal invasiveness in one session, including: direct removal of the tumor and its degradation products; recuperation of the side-effects of the tumor itself, enabling recovery of the visual field loss; retinal reattachment; and prevention of cataracts (by direct lens replacement) [6,7]. Bechrakis et al. demonstrated that resection after neoadjuvant radiotherapy is a promising procedure in the treatment of large intraocular melanoma [6,7].

However, depending on tumor location, it can be necessary to use the optimum dose fall-off (DG) between the gross tumor volume and the macula, as well as the optic nerve, to protect vision acuity. The dose gradient depends on both the precision of the set-up and the radiation technique. Stereotactic radiosurgery (SRS) is already a clinically highly recognized and established treatment modality for cranial or skull-base tumors, as well as for tumors of the eye [8,9]. In the treatment of malignant eye neoplasm, stereotactic radiotherapy allows a contactless preoperative treatment, and thus minimizes secondary symptoms, such as thinning and softening of the sclera, or edema formation. Optimization of dose distribution to irregularly shaped tumors in fixed, immobile areas is achievable with modern medical linear accelerators (LINAC) in the sub-millimeter range [10,11,12]. In the last few years, the therapeutic potential of this method has been increased by further development, from 3D conformal arcs or static-field IMRT to non-coplanar volumetric arc radiation therapy (VMAT), using micro collimators or robotic radiotherapy for small targets [12,13]. Optimization algorithms for dose and beam directions are steadily evolving, and there is great potential for further improvement of the dose distributions [14].

This prospective study of radiosurgery followed by resection was designed to adopt LINAC-based image-guided volumetric arc therapy (IG-VMAT) with micro-multileaf collimator for preoperative radiotherapy for large intraocular melanoma [15]. The primary goal was to incorporate new technical innovations, such as high-resolution 3D image guidance, and continuously surveilled (as fourth dimension) highly conformal radiation dose delivery, with the aim of optimizing both high-dose and low-dose distribution and, depending on tumor localization, the potential of an effective vision-preserving therapy [16].

One of the main strengths of this method is the cone-beam CT-based 6-degrees-of-freedom image guidance and non-coplanar micro collimator-based VMAT, which allows 3D dose conformation. This trial analyzed the feasibility of radiosurgery in large intraocular melanoma, the treatment parameters used and the immediate functional outcomes.

## 2. Materials and Methods

### 2.1. Study Design and Patient Eligibility

This trial is an observational study, based on a prospective registry. The ethics committee of the Medical Faculty of the University of Duisburg–Essen approved the study (21-10377-BO). The research was performed in accordance with the Declaration of Helsinki, and with local relevant guidelines and regulations.

All consecutive patients with stage IIA-IIIB (AJCC) large intraocular melanomas, diagnosed between August 2019 and November 2021, who were amenable to radiosurgery and resection, and who had written, informed consent to participate, were enrolled. Standard brachytherapy with ruthenium-106 (^106^Ru) was not applicable in all cases, as the tumors were not covered by the dose distribution, or due to other tumor-related complications, such as retinal detachment. The following criteria were mandatory for registration: good ECOG performance status (0–1); compatibility with general anesthesia, to allow subsequent surgical resection; and patient consent. The exclusion criteria were: inability to receive magnetic resonance imaging or anesthesia; severe comorbidity; and clinical parameters, such as blindness, painful eyes and no light perception. Eligible patients presented no risk features, such as ring melanoma, broad tumor involvement of the ciliary body (CB), more than 25%, or gross extraocular tumor extension (Table 1) [1]. The COMS original criteria and COMS (Collaborative Ocular Melanoma Study) characteristics for large uveal melanomas were evaluated [17,18].

### 2.2. Treatment Schedule

**Figure 1:**
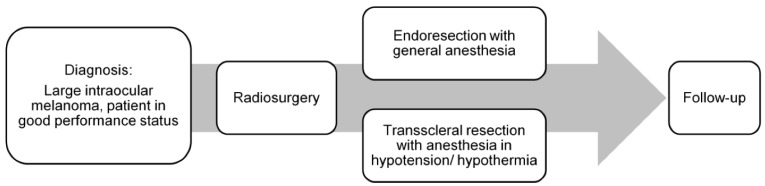
Treatment schedule: image-guided volumetric modulated arc radiosurgery, followed by resection.

The coordination of overall treatment was based on the decision of an interdisciplinary therapy recommendation and its approval. The treatment was divided into two major parts. The first one was CB/CT image-guided, single-fraction volumetric arc (VMAT) SRS, performed in the Department of Radiotherapy, followed by the endoresection (ER) or transscleral resection (TSR) in the Department of Ophthalmology (Figure 1) [4].

### 2.3. Treatment Planning, Part 1: Imaging, Target Definition and Treatment Set-Up

Firstly, treatment planning required accurate evaluation of tumor volume by fundoscopy, ultrasound examination and, if necessary, a fluorescence angiography, including determination of the basal tumor diameter, tumor thickness, localization and distance to the macula and optic disc. 

**Figure 2:**
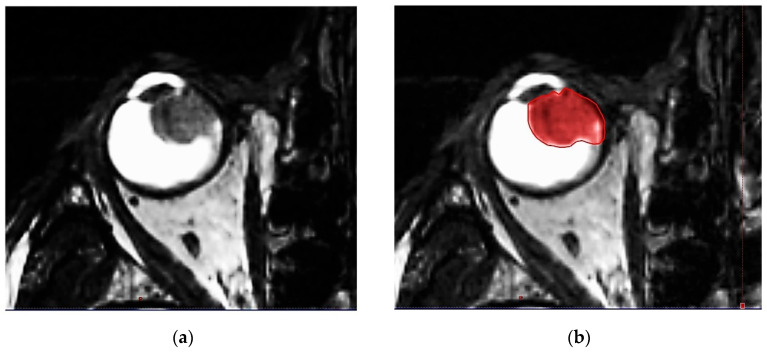

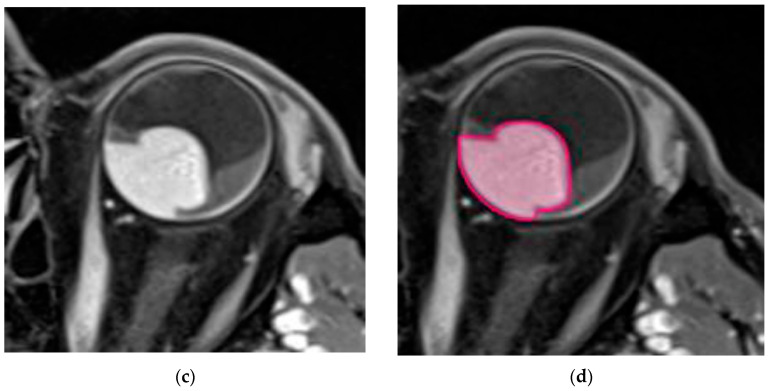
(**a**) Highly prominent choroidal ciliary body melanoma, axial plane, MR T2 ciss sequence, 0.5 mm. (**b**) Highly prominent choroidal ciliary body melanoma, axial plane, MR T2 ciss sequence, 0.5 mm, definition of the uveal melanoma. (**c**) Highly prominent choroidal melanoma, axial plane, MR T1 TSE sequence, bilateral retinal detachment. (**d**) Highly prominent choroidal melanoma, axial plane, MR T1 TSE sequence, bilateral retinal detachment, gross tumor volume (GTV) definition.

IG-VMAT demands an image guidance-based treatment modality. All relevant anatomic features of the patient (eye bulb, lacrimal gland, lens, optic nerve, retina, sclera, contralateral eye structures, brain, hippocampi, etc.) are defined. High 3D spatial accuracy and tissue contrast definition are important features for using IG-VMAT-SRS to its utmost positional exactness [19,20]. A thin-layer MR was acquired, using 0.6–1.0 mm layers with stable, reproducible view fixation (3T MRI scanner). The pre-therapeutic gross tumor volume (GTV) was preferably delineated, based on a contrast-enhanced, fat-saturated T1 sequence (1 mm multiplanar reformation or reconstruction (MPR), and 1–2 mm turbo spin echo (TSE) DIXON technique) in all MR planes (coronal, sagittal and axial). In addition, thin-slice T2 sequences were performed, to check tumor spread and anatomy (Figure 2a–d).

Furthermore, after fabrication of a firm rigid mask [21] (frameless radiosurgery mask system by Brainlab^®^), a thin-slice CT with 1 mm layers was performed for irradiation planning. This was also carried out contrast-enhanced and with stable, horizontal view fixation, analogous to the MRI. The time of CT acquisition was fast. Motion at the time of simulation may affect ideal tumor delineation; thus, standardized viewpoint localization in the centerline was necessary. Ocular motion was mitigated by setting a defined spotlight at the time of simulation, to minimize false rotation of the eye bulb.

The diagnostic orbital MRI examination was integrated into the treatment-planning process by import and rigid registration with the planning CT within the treatment planning software, ECLIPSE (Varian). CT and MRI imaging studies were co-registered, using a rigid match based on ocular structures. For target delineation, an ultra-high-definition pen display, with multi-touch capabilities and a fine-tip stylus, was used [22,23,24]. The gross tumor volume (GTV) was defined as the visible tumor on contrast-enhanced MRI. The planning target volume (PTV) included a margin of 1.5 mm laterally and 2 mm posterior or to the vitreal body around the GTV, that could be reduced to a minimum 0.5 mm towards the optic nerve or 1.0 mm towards the eyelids, if substantial normal tissue sparing was thereby achieved. The volumetric plan was generated in Brainlab Elements (version 3.0). The contours and match results were transferred from Eclipse to Brainlab. The main prescribed dose to the PTV was 22.0 Gy. This dose had to cover, as a minimum dose, at least 99.5% of the PTV (definition of D99.5%: dose, which 99.5% of the PTV receives), i.e., the D99.5% for the PTV had to be ≥22.0 Gy. The accuracy of the dose calculation depended on the resolution of the dose grid. The grid size used for the final irradiation plan did not exceed 0.63 mm [25].

### 2.4. Treatment Planning, Part 2: External Beam Planning, Dose Metrics and Quality Control

For this treatment, a TrueBeam^®^ SRS medical LINAC (Varian Medical Systems, Palo Alto, CA, USA) with Novalis^®^ Radiosurgery (BrainLab, Munich, Germany), provided with a high-definition multileaf collimator of 2.5 mm width, was used. For minimizing treatment time, the flattening-filter-free (FFF) beam function, with 6 MV and with dose rates (up to 1400 MU/min), was applied [26].

**Figure 3:**
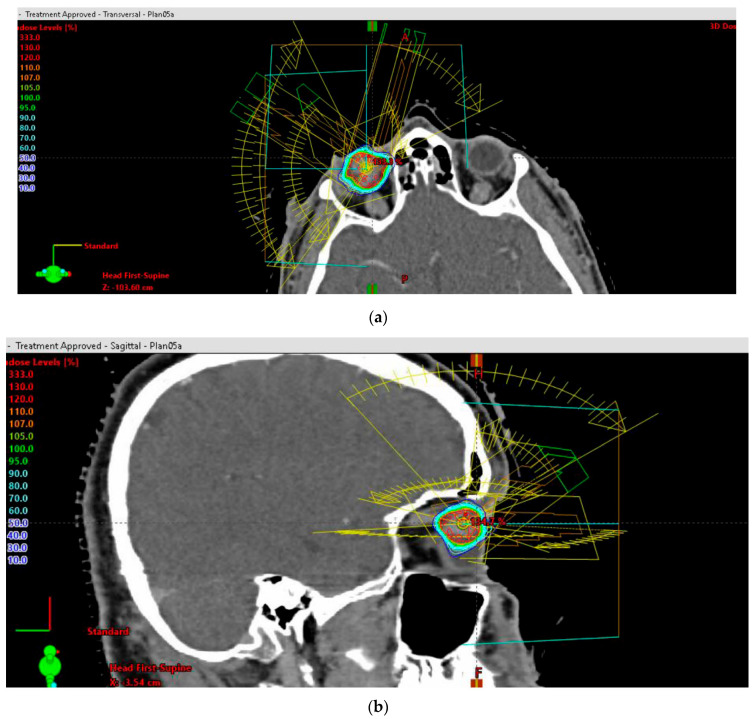
(**a**) Stereotactic radiosurgery (SRS) by image-guided (IG) volumetric modulated arc therapy (VMAT) for patients with unfavorable-risk, large intraocular melanoma: representative 3D beam arrangements—axial plane. (**b**) Stereotactic radiosurgery (SRS) by image-guided (IG) volumetric modulated arc therapy (VMAT) for patients with unfavorable-risk, large intraocular melanoma: representative 3D beam arrangements—sagittal plane.

Three-dimensional beam arrangements were set and premeditated for each case, to deliver highly conformal prescription dose distributions. Four or more non-opposing, non-coplanar beams were selected. Entry and exit fields were individually optimized, and were required not to pass through the skull base, brain or body trunk below the upper borders of the breasts. The field aperture dimension corresponded to the beam’s eye view (BEV) projection of the PTV. The field arrangements (entrance–exit fields) were set up so as not to pass through sensitive structures. The main couch angles, as well as the gantry starting and stopping angles, were set up beneath the skull base, in accordance with the other OARs, to complete the trajectory optimization. A representative beam arrangement is shown in Figure 3a,b.

The goals for ocular IG-VMAT planning were, that the prescribed dose (PD) should cover 99.5% of the PTV, and that the dose gradient around the target should be maximized, subject to sparing the brain. Under the above conditions, the maximum dose within the PTV reached 140–145%. 

The cranial SRS planning system by Brainlab Elements was used (version 3.0). A special workflow, based on the 4Pi algorithm for optimizing the radiation angles and couch positions, allowed complex target volumes to be treated [27].

The following conformity measures for the planned dose distribution around the PTV were used: the ratio of the 50% prescription isodose volume (PIV) (ml) to the volume of the PTV (ml) (R_50%_); the inverse Paddick conformity index (CI), comparing the prescription isodose volume and the target volume with their geometrical overlap; the gradient index (GI), comparing the 50% and 100% isodose volumes [25]; and the dose-gradient index (DGI) [28]. Beneath these isotropic measures, the dose fall-off gradient beyond the PTV, towards the most critical normal tissue structure, e.g., the optic disc, was recorded. The absolute mean and maximum dose limits for critical structures were measured.

As a standard procedure in medical physics, plan verification was carried out, using Monte Carlo simulations with the ProSoma^®^ planning system [29].

### 2.5. Pre-Treatment Preparation, Immobilization Devices and Technique

Immediately before the planned IG-VMAT-SRS, retrobulbar anesthesia (RBA) was applied to the affected eye. The goal was a complete immobilization of the eye bulb for the duration of the next 1.5–2 hours. A pre-treatment CT scan was performed directly thereafter, to determine the new eye bulb and tumor positions, and how they differed from the original zero position within the planning CT. The exact tilt angle was measured. The standard 6 DoF (6-degrees-of-freedom) Varian couch allowed for tilt (rotation about the cross-table axes) and roll (rotation about the table’s long axis) correction of plus–minus 3 degrees, in addition to the coach rotation for yaw angle correction. We developed special head-positioning systems to compensate for a larger rotation of the eye bulb, due to the RBA. For enabling a larger tilt or roll deviation, we used manufactured wedges for cranial positioning of 5, 8 or 11 degrees in cranio–caudal, caudo–cranial, right–left and left–right directions. This allowed many more degrees of freedom for the head, and an eye alignment in the center line. These wedges were an additional, customized tablet between the 6-degrees-of-freedom positioning table at the linear accelerator and the base plate of the mask-fixation system. The yaw angle was corrected by coach rotation around the isocenter. The eyelids were widened by sterile, non-metallic lid-spreaders, in order to pull the eyelids out of the high-dose area. The cornea and conjunctiva were covered with a long-lasting eye gel that prevented drying out.

### 2.6. Treatment, Image Guidance

After immobilization in the treatment room, a cone-beam CT (CBCT) was acquired online with 800–1000 mAs and 50 mL i.v. contrast medium bolus. In this way, an improved tumor contrast was obtained (Figure S1). The final eye-based match was then performed by the radiation oncologist, using the contouring module of the Eclipse planning system, using 6 degrees approximation. Landmarks for matching were the visible intraocular contrasted tumor, the insertion of the optic nerve, the lens and the shape of the bulbus. The whole radiation session was continuously surveilled online, using an optical surface imaging system controlling the eye position (presenting the role of time as the fourth dimension). For this purpose, we used a high-precision video system to document the immobility of the eye bulb. For safety reasons, due to the application of local anesthesia, pulse and oxygen were also monitored during the session. A qualified medical physicist was present for the set-up and motion review. The radiation oncologist performed and approved the image guidance, monitored immobilization of the eye, and was also present at the whole treatment fraction.

### 2.7. Histopathology and Tumor Characterization

After resection, all tumors underwent histopathological processing. The diagnosis of uveal melanoma was routinely confirmed with the detection of a GNA11/GNAQ mutation analysis in cases that were not absolutely clear (by Sanger sequencing of exons 4 and 5 in the DNA of the tumor sample). In addition, in some patients (depending on personal preferences and written consent) prognostic testing (determination of chromosome 3 status) was performed by genotyping 8 STR loci on chromosome 3 from blood and tumor cells.

### 2.8. Follow-Up

Follow-up after IG-VMAT-SRS was daily for 10–20 days; therapy took place on an inpatient basis, due to the subsequent resection. This was followed by a 3-to-6-monthly presentation during the first year. Side-effects and eye functions—defined on the basis of a 5-point Common Terminology Criteria for Adverse Events (CTCAE) Version 5.0 assessment score after radiosurgery, as follows: (i) visual acuity; (ii) inflammation, including keratitis, uveitis, watery eyes or corneal ulcer; (iii) eye pain; (iv) optic nerve disorder, including papilledema, and (v) other ocular side-effects (hemorrhage, extraocular muscle paresis, eyelid dysfunction, glaucoma, flashing lights, photophobia, periorbital edema) [30]—were quantified immediately after SRS: on days 1–5 before surgery; on the day, at discharge after surgery and first interdisciplinary follow-up control; at 3–6 months; 12 months; and at the last follow-up. We divided the follow-up into several stages: (i) early, after SRS and before surgery intervention; (ii) early, after SRS and after surgery; (iii) early interval, 3–9 months after SRS and surgery; (iv) 9–18 months; and (v) more than 18 months after SRS and surgery.

## 3. Results

A total of 50 patients completed the baseline survey and at least one post-baseline survey in the time period from August 2019 to November 2021. The median age was 55 years (range 26–84 years). Twenty-four patients were females. Patient and tumor characteristics are shown in Table 1.

**Figure 4:**
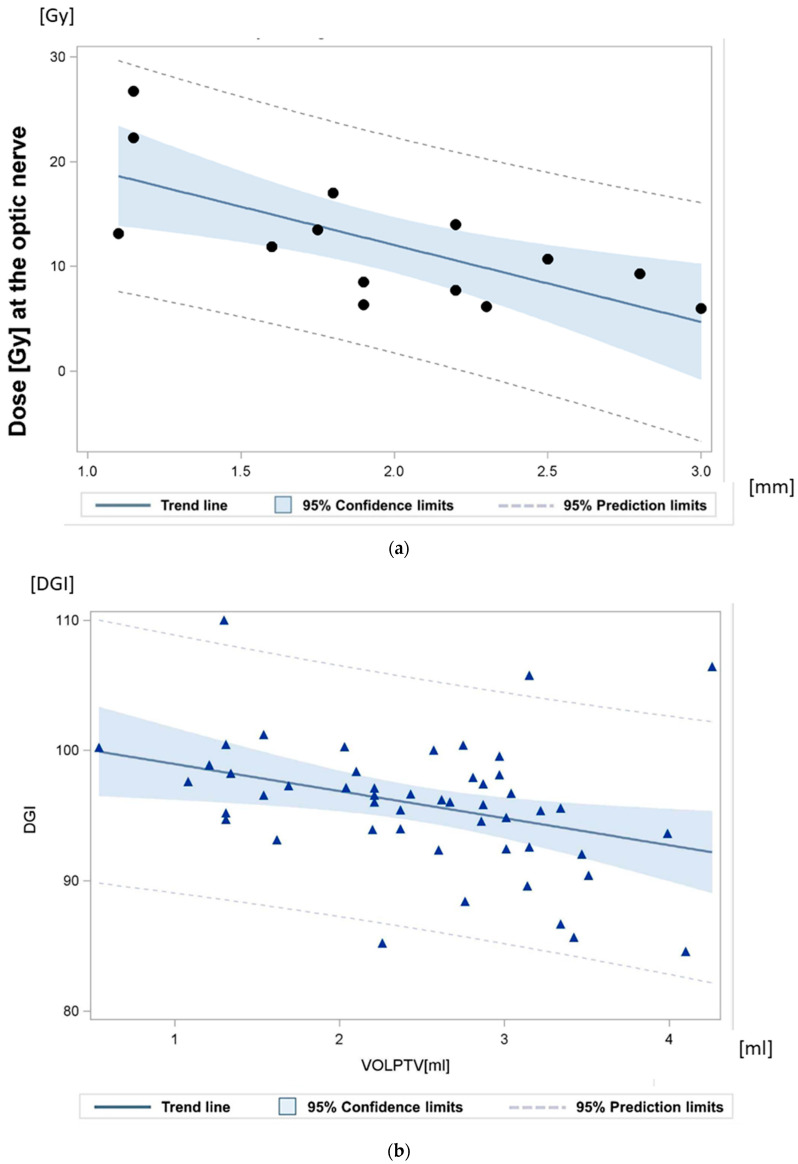
(**a**) Relationship between the maximum dose at the optic nerve, and the minimum distance between the gross tumor and the optic disc. The achieved dose gradient was 7.3 Gy/mm +/− 2.1 Gy/mm. “•” Each sign representing the radiation plan of one patient. (**b**) Dose-gradient index (DGI) quantifying the dose fall-off: the mean dose-gradient index (DGI) for an equivalent sphere equaled 121.20 (120.00–122.64). The calculated DGI for the irregular target volume amounted to 95.86 (84.5–110.01). “
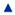
” Each sign representing one radiation plan.

One of the most important quality parameters of the radiation plans was the protection of the optical axis, optic nerve and macula (Figure 4a). The mean gross tumor target volume (GTV) was 1.19 mL (0.40–2.94 mL), and the mean tumor thickness (TT) was 11.15 mm (8.0–15.50 mm). The largest basal diameter (LBD) measured was 15.11 mm in mean (range: 7.6–21.90 mm), and the mean small basal diameter (SBD) was 13.58 mm (range: 6.31–20.40 mm). In 33 patients (66.0%), the tumor was located both in front of and behind the equator. Fifteen patients (30.0%) showed tumor growth within 1–3 mm close to the optic disc. In addition, in 15 patients the tumor extended to the ciliary body (CB), with a gross infiltration of less than 25%. Retinal detachment in two or more quadrants was present in 25 patients, and in only one quadrant in 15 patients. All patients (100%) fulfilled the COMS original criteria, and 92% met the classic COMS (Collaborative Ocular Melanoma Study) criteria for large uveal melanomas [17,18].

**Figure 5:**
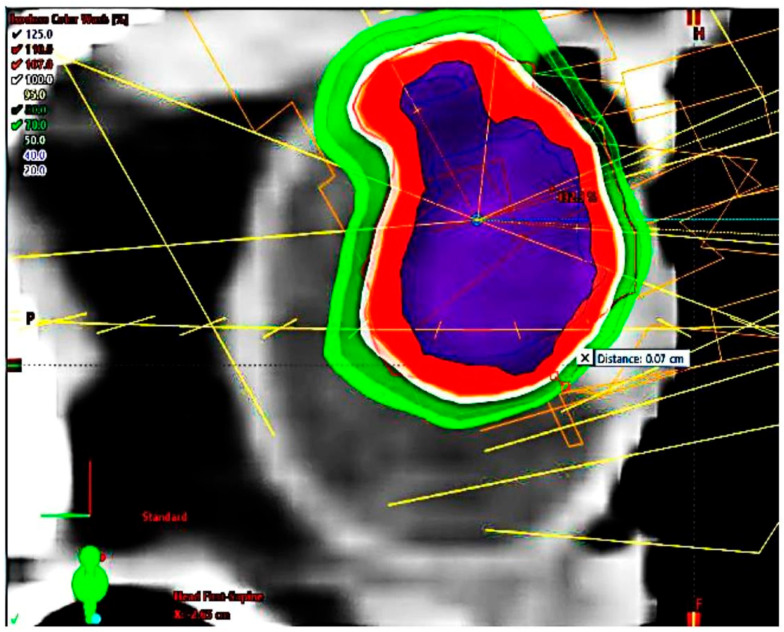
Highly prominent choroidal ciliary body melanoma, dose distribution, sagittal plane, maximal sparing of cornea and eyelids.

The maximum dose gradient (DG) toward the most critical normal tissue between isodose D_100%_ and isodose D_50%_ was 1.5 mm on transverse planes and 1.2 mm on coronal planes. The achievable DG between isodose D_100%_ and isodose D_70%_ towards the most critical structure at risk was 0.75 mm. For central tumors, the actually achieved gradients between D100% and D50% toward the optic nerve were in mean 1.7 mm (95% CI: 1.5 mm–2.0 mm). The actually achieved gradients toward the lids, if they were close to the planning target volume, were 1.7 mm. In patients with tumors close to the optic nerve (0.7–3.0 mm), the distances between the GTV and the optic nerve were quantified (Figure 4a and Figure 5).

The prescription dose D_99.5%_ for the PTV ranged from 19.01–25.01 Gy, mean 22.12 Gy. The mean dose to the PTV over all patients was 26.67 Gy (24.72–30.53). The mean number of selected arcs amounted to 5 (4–12), all arranged with minimized brain entrance or exit fields (Figure S2). The mean brain dose was only 0.14 Gy (0.04–0.26 Gy). The radiation dose to the 1cc of brain with the highest dose exposure was in mean 1.7 Gy (0.65–2.8). The ratio (R_50%_) between the 50% isodose volume (PIV50%) [7.55 mL (2.70–16.00)] and the PTV was in mean 3.12 (range: 2.00–5.00), and the mean ratio between the ESD of the PIV50% and the ESD of the PTV was 1.457 (range: 1.27–1.70) on average. The ratio of the ESD of the PIV100% to the ESD of the PTV averaged 0.962 (0.89–1.10). The mean conformity index (CI) was 1.2 (1.09–1.55); the mean gradient index (GI) amounted to 2.6 (2.35–3.38). The mean dose-gradient index (DGI) for an equivalent sphere equaled 121.20 (120.00–122.64). The real DGI for the irregular target volume amounted to 95.86 (84.5–110.01) (Figure 4b).

Therapy was performed in a relatively short time. The whole SRS treatment time, with patient onboard, averaged 17 min (14–25 min).

The mean follow-up period was 18 months (7.2–36 months). During follow-up, no local recurrences were observed. Two patients developed hepatic metastasis without local recurrence. The maximum dose to the optic nerve was 7.77 Gy (range: 1–26.74 Gy) on average, and was only more than 10 Gy for eyes with a tumor-to-optic-nerve distance of less than 1.5 mm.

Shortly after radiosurgery (mean 5 days, range 1–13 days), there were no side-effects, or only very mild side-effects, related to radiotherapy on the 5-point CTC assessment scale 1–5 (Table 2). Four patients described mild pain. Eye pain for post-intervention was attributed to the RBA, and it was also effectively and immediately interrupted with NSAID medication. One patient reported transient floaters, characterized by spots in front of the eye; the patient had already presented with retinal detachment, which did not worsen after the IG-VMAT-SRS. Immediately after resection, in most patients there were limited side-effects: all patients had the common post-operative conjunctival edema (in the area of the anterior segment of the eye). This redeveloped properly. One patient evolved corneal edema; by intensive eye treatment, it was possible to treat this effectively. In the early post-operative follow-up, 1 patient developed ocular hypotension; this had to be corrected by surgery. Another, more common, side-effect was the occurrence of edema, e.g., macular edema in the posterior segment of the eye: depending on the manifestation of edema, parabulbar or intravitreal cortisone treatment usually was effective. Several patients showed an elevated intraocular pressure (IOP) after resection. The IOP was adjustable, and lowered with intensive medication. One patient developed a new onset of retinal detachment during the course, under silicone oil; by early intervention, this was treated.

During follow-up, the bulb was preserved in 98% of all cases. One 76-year-old patient, with a tumor in the anterior segment of the eye, showed a spontaneous total hyphema of the anterior chamber 10 days after IG-VMAT and endoresection, so that the eye had to be enucleated; in this patient, acetylsalicylic acid was discontinued a minimum 8 days prior and after to surgery, as it was administered only as prophylaxis for transient cardiac arrythmia; whether other risk factors of bleeding were present, could not be established. The need for silicone oil removal caused at least one further pre-planned surgical treatment. The patient with the largest initial tumor volume (2.94 mL) started to develop a scleromalacia, at which point it could still be treated conservatively.

Fifty percent of patients had a follow-up longer than 18 months. Fourteen patients showed a prolonged or persisting macular edema in the last follow-up. These patients received a median macula dose of 13.82 Gy (4.08–24.4 Gy), due to tumor localization.

In the mean follow-up of 18 months (7.2–36), 90% of all patients showed a vital optic nerve disc in the last follow-up.

In all patients with tumors located more than 0.7–1 mm apart from the optic nerve, partial volume sparing of the optic nerve head (ONH) was achieved; only two showed incipient optic nerve pallor as a sign of developing ONH atrophy.

In 7 patients, the tumor directly extended to the ONH; in these cases, the dose could not be reduced in the part of the optic nerve directly adjacent to the tumor. One of these patients developed optic nerve atrophy at 12 months, with residual light perception still preserved at 20 months. Two showed an onset of pallor texture of the OND in the last follow-up of 34.97 and 36.13 months.

In at least 32 of all patients with a longer follow-up, it was already possible to make reliable statements about the visual outcome. In 31 patients, the visual acuity was above 0.1, in 21 s.c. (sine correctione). Eight patients demonstrated an improvement of visual acuity. For the remaining cohort, further treatments were necessary, or no final quantifiable measurement was documented because, for example, silicone oil had not yet been removed.

## 4. Discussion

The present prospective observational study investigated the feasibility and efficacy of single-dose IG-VMAT-SRS followed by resection in patients with stage IIA-IIIB high-risk large intraocular melanoma.

SRS of the eye represents an effective but challenging intervention, due to the inherent rotational movability of the eye, the irregularity of the target volume and the close vicinity of organs at risk [12,31,32]. The purpose of the present analysis was to report procedure and realization, dosimetric plan quality, overall treatment time and early outcome of SRS eye plans followed by resection in high-risk large uveal melanoma.

Endoresection and transscleral resection per se are very effective procedures in treatment of uveal melanoma. Nevertheless, Damato et al. described the main risk factors for local tumor failure after surgery as monotherapy, especially in large uveal melanoma. Recurrence at 4 years varied, from low rates below 10% in smaller uveal melanoma, up to 57% if there were more than two defined risk factors. These included: an R1 resection; tumor distance, to the optic disc, below 1 disc diameter; epitheloid histopathology; tumor base greater than, or equal to, 16 mm; and lack of adjuvant brachytherapy [5]. Kim et al. 2002 also described their experience with high-risk uveal melanoma and uncommon relapse rates. The authors stated that, after a sole surgical approach, uncontiguous tumor recurrence primarily only occurred in eyes with aggressively growing, large uveal melanomas [33].

Furthermore, a local failure significantly increased the risk of secondary metastatic uveal melanoma [34].

A first systematic review and meta-analysis of SRS as monotherapy provided the first positive evidence, with excellent outcomes [9,12]. Parker et al. also showed, in a recent meta-analysis, local tumor control rates of 96% with radiosurgery alone [13]. Gamma-knife radiosurgery demonstrated high efficacy as monotherapy in the treatment of intraocular melanoma and metastases [13]. Preliminary data are also available for CyberKnife^®^. Eibl-Lindner and Mor delivered the first information on CyberKnife^®^ monotherapy, describing a functional improvement in residual visual acuity of 0.3, or higher, in 31% of cases after treatment [35,36]. However, Biltekin and Yazici estimated secondary malignancy risk as being almost two times higher in robotic SRS than in LINAC-based techniques, with a more limited multitude of beam angles [37]. Weber et al. compared proton and SRS photon plans, concluding in 2005—with the techniques available at that time—that there was a similar level of dose conformation in comparison to static field IMRT or conformal arc photon therapy with uniform intensity fields [31]. According to Slopsema et al. the lateral penumbra in proton beams varies between 0.9 and 2.2 mm. This distal dose gradient, from the 90% to the 10% isodose, ranged from 0.7 mm–3.2 mm for the different centers, but most groups used an additional depth margin of 2.5 mm at both the distal and proximal end of the gross tumor volume, to account for uncertainties [35,38,39,40,41]. Gragoudas reported a 40% incidence of radiation-induced maculopathy in tumors more than 1 disc diameter from the macula with proton therapy [42]. The precision achieved in SRS depends on the accuracy of the target volume definition, the required PTV margin around the GTV, and the dose gradients beyond the PTV. Slightly smaller PTV margins were used by CyberKnife groups using 1.0 mm radial PTV margins and 2.0 mm for posterior treatment margins [35,36]. Wösle et al. achieved maximum dose gradients around the CTV of about ~23%/mm, with a dynamic conformal arc technique [43]. We treated patients with margins of about 0.5–1.0 mm towards the optic nerve, for tumors closer than 3.0 mm to the optic nerve. This precision was achieved by direct visualization of the optic nerve and the tumor by cone beam CT, and immobilization of the eye by retrobulbar anesthesia. For these ONH-close tumors, a dose gradient from the PTV margin to the 50% isodose of 1.2 mm on coronal and 1.5 mm on axial planes was achieved; these were very competitive, in comparison to other techniques.

Radiosurgery performed with LINAC showed similar early outcomes to other percutaneous radiation techniques for intraocular melanoma. Ciernik et al. (2018) analyzed the difference of a combination of dynamic conformal arcs (DCA) complemented with multiple non-coplanar static intensity-modulated (IMRT) fields (DCA-IMRT), and volumetric modulated arc therapy (VMAT). They reached conformity indices of 1.24 (1.05–1.77) for DCA-IMRT and 1.31 (1.11–1.50) for VMAT; there, the mean doses applied to the ipsilateral optic nerve were 7.3 Gy (0.4 Gy–22.2 Gy) with DCA-IMRT, and 13.4 Gy (8.2 Gy–17.4 Gy) with VMAT. The beam-on time for DCA-IMRT was 3.2 (±0.4) minutes, and 2.9 (±0.25) minutes for VMAT. [12] Georg et al. (2003) also evaluated the importance of a micro multileaf-collimator (mMLC) on LINAC-based stereotactic radiotherapy (SRS) of uveal melanoma, by comparing circular arc, static conformal, dynamic arc, and intensity-modulated SRS. [44] Wösle et al. treated uveal melanomas with PTVs, ranging from 0.42 to 3.37 cm^3^; there, the conformity index (CI) was 1.25 ± 0.15, and the homogeneity index (HI) was 0.08 ± 0.02. In order to characterize the differing gradients to organs at risk, the authors defined a mean dose gradient as the difference quotient averaged over all spatial directions—called ‘spatially averaged dose gradients’ (SADG) [43].

All these studies to date have been limited by the lack of resection, or by the precise onboard 3D or 4D image guidance, and the consequent limited treatment and toxicity control.

In large uveal melanoma, Suesskind et al. found that SRS combined with tumor resection could be associated with fewer local tumor complications than SRS monotherapy [45]. Here, the advantages of both technologies can be combined: on the one hand, SRS devitalizes the tumor; on the other hand, the noxae that large uveal melanoma have already caused, and that can be further triggered by tumor remnants, can be surgically repaired. Potential severe sequelae may be limited by the use of both treatment modalities [9,46,47]. This study went even further: it used the application method of high-precision image guidance—SRS. Especially for large intraocular tumors, a combined treatment modality seems to be an excellent treatment approach, as the eye is in danger of being lost in a very short time-period, due to the tumor itself [45,48,49,50,51]. Here, we could also see that single-dose IG-VMAT-SRS, with retrobulbar anesthesia followed by resection, showed very good and satisfactory results during the first follow-up, with respect to freedom from tumor recurrence in high-risk large uveal melanoma. No adverse events, or very limited adverse events, were observed.

The combination of the two modalities—SRS and tumor resection—led to a significant improvement in early outcome [15,47,48].

Romano et al. also described, in a systematic review, vitreoretinal surgical approaches, in order to prevent a toxic tumor syndrome of the eye [52].

Enucleation is related to a large tumor size, tumor thickness, proximity of posterior tumor margin to the optic disc, high intraocular pressure, and large degree of retinal detachment at treatment time [53,54]. Endoresection and transscleral resection showed the potential to minimize the side-effects of the toxic tumor syndrome [55].

In SRS, the optic nerve tolerates single doses of 12–15 Gy with a risk of approximately 10% of radiation-induced optic neuropathy [56,57]. Due to the high conformity, steep dose gradients can be obtained towards the optic nerve. If there is only a peripheral unilateral tumor extension to the optic nerve (without immediate infiltration of the optic disc, e.g., colliculus nervi optici or the porus opticus), the contralateral part may be spared. Here, in 14% of cases, the tumor directly extended to the ONH. In this case, the dose could be reduced only at the contralateral, rather than the part of the optic nerve adjacent to the uveal melanoma. The significance of the dose maximum, in partial volume irradiation at the ONH and the optic nerve, has not yet been systematically evaluated. For tumors close to the papilla (up to more than 1.0–1.5 mm from the central papilla), and tumors which do not grow in a circumpapillary manner, the whole optic nerve and ONH can be spared by IG-VMAT SRS.

Here, we used a standard dosage for SRS of tumor lesions according to an established concept, with the aim of permanent local tumor ablation. The mean applied dose to the GTV was 27.31 Gy (24–31.37 Gy) with a prescription dose of 22 Gy to the PTV. Other colleagues used single doses ranging from 20 Gy (Eibl-Lindner et al.) through 21 Gy (Joye et al., Özcan et al., Arnett et al. and Mor et al.) up to 25 Gy (Biewald et al.) [15,35,36,58,59,60].

Suesskind 2013 et al. also applied 25 Gy to the tumor, without a recurrence [45,61]. With SRS by robotic radiosurgery, the 3-year control rates were 87.4% with 18–22 Gy; however, tumor-related side-effects (including hemorrhage or glaucoma) also occurred in 30% [35]. Béliveau-Nadeau et al. described, in their work, the robotic surgery CyberKnife at the Center Hospitalier de l’Université de Montréal (CHUM), at the University of Montreal, Canada [62].

The colleagues used SRS at their Center in juxtapapillary tumors, to avoid a high dose with brachytherapy on the optic nerve, and thus to preserve a residual visual function. The conformity index (CI) was below 1.5 [62].

Wösle et al. reached a conformity index (CI) of 1.25 ± 0.15, and a homogeneity index (HI) of 0.08 ± 0.02, by means of HybridArc™ [43].

Nevertheless, the approach was novel, as we used an advanced VMAT-technique and a soft tissue contrast-sensitive 3D image guidance-based cone-beam CT for these large uveal melanomas, that can easily be detected on these images. Proton centers and CyberKnife used stereoscopic X-ray lines for image guidance, that made the application of clips necessary. The relation of the tumor to the clips and structures at high risk, such as the optic nerve, had to be determined, and the clips were used as markers for image guidance. In this study, the eyes were immobilized by retrobulbar anesthesia, that made treatment delivery over about 17 minutes largely independent of patient compliance, which is important for treatments without anesthesia [63]. An advantage of cone-beam-based image guidance is the direct visualization of large uveal melanomas and the optic nerve. The technical adaptation of IG-VMAT-SRS shows good feasibility, and very good early outcome data.

The present study used high-resolution MRI, as well as the fundus views for target volume definition. MRI is a very important modality for determining the gross target volume, because of its superior soft-tissue contrast and spatial resolution [64]. The lesion was evaluated in the axial, sagittal and coronal planes. However, if a lesion was located in the ciliary body, in the posterior wall region of the eye bulb, or in close proximity to the optic nerve, additional sagittal oblique images were obtained for an optimal evaluation. Ocular sonography is another important pre-treatment study, in order to exclude extrascleral extension and primary tumor diameters.

The strengths of this study lie in the use of high-resolution MRI for treatment planning, advanced non-coplanar VMAT optimization procedures, retrobulbar anesthesia and cone-beam CT-based image guidance. The dose to the macula and optic nerve can be calculated prior to therapy application; thus, the risk to visual acuity can also be estimated. Tumors very close to the papilla can be treated with SRS, with an optimal gradient of up to 1.5 mm (D_max100%_-D_max50%_ on axial planes). The optic nerve may also be partially spared, for tumors located closer to the ONH, when there is no walling of the ONH. Prior treatment selection entailing a thorough risk assessment is mandatory.

Furthermore, it may be confirmed here, that not only transscleral resection but also endoresection requires great vigilance, in avoiding post-operative bleeding. SRS is shown to be a procedure that is safe for the patient and does not require application of tantalum clips.

Some limitations should be taken into account. Firstly, the mean follow-up period of 18 months was too short to make a definitive statement on the long-term outcome. Furthermore, for transscleral resection, patients had to be clinically in a good performance status, without higher comorbidities. The theory that combined treatment of modern image-guided LINAC SRS and resection leads to greater effects, compared to radiotherapy alone, needs to be tested in comparative studies, with long-term follow-up.

## 5. Conclusion

Overall, this experience demonstrated that 4D image-guided volumetric modulated arc radiosurgery (IG-VMAT-SRS) followed by resection has good tolerability and effectiveness in the primary follow-up. Gradients towards critical organs at risk are very competitive with other radiotherapy techniques.

## Figures and Tables

**Table 1:** Main Inclusion and Exclusion Criteria, Patient and tumor characteristics.

**Obligatory Inclusion and Exclusion Criteria for Combined Radiosurgery and Resection**	
**Inclusion criteria**	
Standard brachytherapy with Ruthenium-106 (^106^Ru) plaque	not applicable
Tumor thickness	≥8.00 mm
Performance status (ECOG)	0–1
Magnetic resonance imaging	obligatory
For endoresection, compatibility with general anesthesia	obligatory
For transscleral resection, compatibility with general anesthesia in hypotension	obligatory
**Exclusion criteria**	
Complete loss of visual acuity	No light perception (Lux defect)
Localization	Ring melanoma
Tumor involvement of the ciliary body	Broad involvement of the ciliary body, ≥25% of the ciliary body
Extraocular tumor extension	Present
Anticoagulation or antiplatelet therapy	If this cannot be discontinued perioperatively
**Patient and tumor characteristics**	
**Patient and tumor characteristics**	**Number of Patients**
**Histology**	
Spindle cell (>90% spindle cells)	26
Mixed (>10% epithelioid cells and <90% spindle cells)	15
Epithelioid (>90% epithelioid cells)	7
Pleomorph cells	2
**Ki67%**	
No expression	13
Low expression	27
Intermediate high expression	10
High expression	0
**BAP1 Immunohistochemistry (IHC)—nuclear staining**	
BAP1 negative	15
BAP1 IHC nuclear positivity: low	9
BAP1 IHC nuclear positivity: high	26
**HMB45**	
Positive	19
Not determined or negative	31
**Monosomy for chromosome 3**	
Yes	12
No	13
Denial of the determination, or not determined	25
**cT-category**	
cT2a	4
cT2b	1
cT3a	23
cT3b	5
cT4a	10
cT4b	7
**cN/M-category**	
cN0/cM0	50
**AJCC stage—8th edition**	
IIA	4
IIB	24
IIIA	15
IIIB	7
**Resection modality**	
Transscleral Resection	11
Endoresection	39
**Age**	**Median and Range**
Median	55 years
Range	26–84 years

**Table 2:** Classification of outcome according to Common Terminology Criteria for Adverse Events (CTCAE).

**Adverse Events within 12 Days after Radiosurgery, and before Resection according to CTCAE Version 5.0**					
**CTC Score immediate after SRS**	**CTC 0**	**CTC I**	**CTC II**	**CTC III**	**CTC IV**
1. Visual acuity	50	0	0	0	0
2. Eye pain	46	4	0	0	0
3. Inflammation	48	2	0	0	0
4. Optic nerve disorder, including papilledema	50	0	0	0	0
5. Other ocular side-effects: hemorrhage; extraocular muscle paresis; eyelid function disorder; glaucoma; periorbital edema	49	1	0	0	0
**Adverse Events within 30 days after resection according to CTCAE Version 5.0**					
**CTC Score:** **After resection**	**Not yet assessable, but light perception verifiable**				
1. Visual acuity	50				
**CTC Score:** **After resection**	**CTC 0–I**	**CTC II**	**CTC III**	**CTC IV**	
2. Eye pain	43	7	0	0	
3. Inflammation	44	6	0	0	
4. Other ocular side-effects: hemorrhage; extraocular muscle paresis; eyelid function disorder; glaucoma; periorbital edema	40	7	1	1	
**Adverse Events more than 30 days after radiosurgery and resection, up to the last follow-up (mean 18 months) according to CTCAE, Version 5.0**					
**CTC Score: Last follow-up**	**Visual acuity improvement**	**idem**	**I**	II and N(Y)Q*	
1. Visual acuity	8	10	14	17*	1*^/^**
**CTC Score: Last follow-up**	**CTC 0**	**CTC I**	**CTC II**	**CTC III–IV**	**N(Y)Q**
2. Eye pain	48	1	0	0	1**
3. Inflammation	46	3	0	0	1**
4. Optic nerve disorder, including papilledema	45	0	3	1	1**
5. Macular edema	24	7	7	0	11*/1**
6. Other ocular side-effects: hemorrhage; extraocular muscle paresis; eyelid function disorder; glaucoma; periorbital edema	39	5	4	1**	0

* not, or not yet, quantifiable (N(Y)Q); ** secondary enucleation due to hemorrhage.

## Data Availability

The data presented in this study are available on request from the corresponding author.

## Supplementary Materials

The following supporting information can be downloaded at: https://www.mdpi.com/article/10.3390/cancers14194729/s1, Figure S1: LINAC cone-beam computed tomography (CBCT), control CBCT scan onboard with intravenous contrast agent after retrobulbar anaesthesia before SRS; visualization of isodose lines; Figure S2: Treatment plan showing the field alignment and the low dose distribution. The brain is completely blocked to irradiation entrance or exit beams.
