# Supplementary material for: Feasibility, Method and Early Outcome of Image-Guided Volumetric Modulated Arc Radiosurgery Followed by Resection for AJCC Stage IIA–IIIB High-Risk Large Intraocular Melanoma
Source: Cancers (Basel). 2022 Sep 28;14(19):4729. doi: 10.3390/cancers14194729 (PMC9562629; doi:10.3390/cancers14194729)
Supplement: Supplementary file 1 [file cancers-14-04729-s001.zip › cancers-1906929-supplementary.pdf]

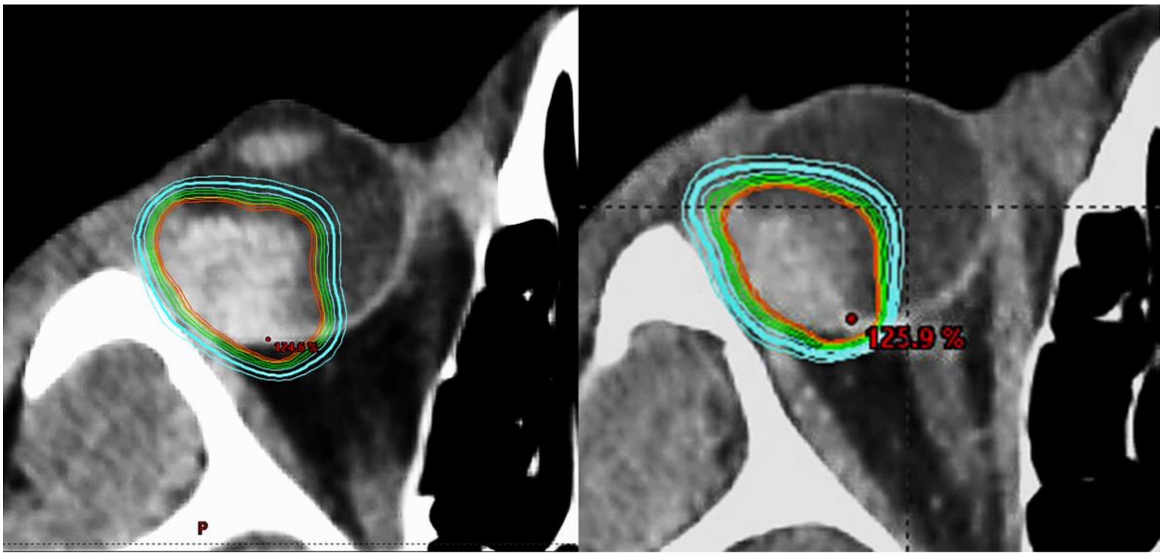

Figure S1. LINAC cone-beam computed tomography (CBCT), control CBCT scan onboard with intravenous contrast agent after retrobulbar anaesthesia before SRS; visualization of isodose lines.

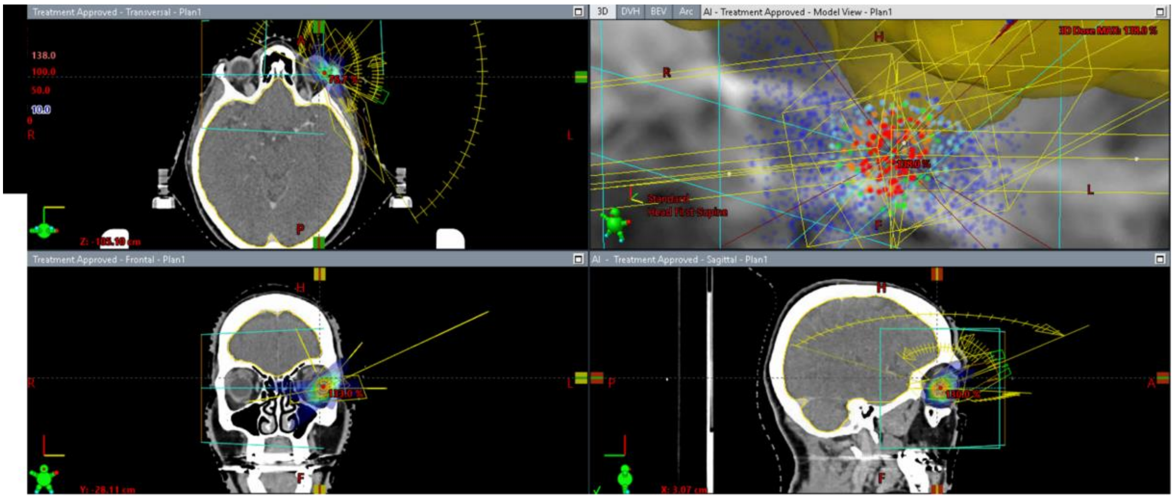

Figure S2. Treatment plan showing the field alignment and the low dose distribution. The brain is completely blocked to irradiation entrance or exit beams.
